# Effect of Steel Fiber Content on the Mesoscopic Damage Mechanism of Cemented Gangue Backfill

**DOI:** 10.3390/ma19153217

**Published:** 2026-07-28

**Authors:** Furong Wang, Xuehua Li, Shenggen Cao, Kaifei Wang, Chiyuan Che, Yang Liu, Yi Li

**Affiliations:** 1 School of Mines, China University of Mining and Technology, Xuzhou 221116, China; 2 State Key Laboratory for Fine Exploration and Intelligent Development of Coal Resources, China University of Mining and Technology, Xuzhou 221116, China

**Keywords:** cemented gangue backfill, PFC2D numerical simulation, steel fiber reinforcement, mesoscopic damage mechanism, crack evolution

## Abstract

To overcome the limitations of conventional numerical simulations of cemented gangue backfill (CGB), this study developed a refined PFC2D model that incorporates the actual particle size distributions of coal gangue and river sand. Randomly distributed steel fibers were generated using FISH programming. Based on uniaxial compression tests and scanning electron microscopy (SEM) observations, the influence of steel fibers on the mesoscopic damage mechanism of CGB is systematically investigated. The results indicate that: (1) the refined model significantly improves the reliability of numerical simulations, accurately reproducing stress concentration within coarse aggregates and the steel fiber “bridging effect”; (2) a steel fiber volume fraction of 0.8% optimizes force chain distribution and suppresses crack propagation, promoting a transition in failure mode from brittle shear failure to ductile compressive–extrusion failure mode, with the peak strength and residual strength increased by 23.7% and 40.2%, respectively, compared with the fiber-free specimen; (3) PFC simulations reveal that steel fibers markedly retard damage accumulation by modifying the force chain network and crack propagation paths; and (4) SEM analysis demonstrates that steel fibers enhance the toughening effect through the interfacial transition zone, whereas excessive fiber content (1.2%) leads to fiber agglomeration and a 62.5% increase in porosity, resulting in performance deterioration. This study provides a robust theoretical framework for gradation reconstruction and refined fiber modeling in the design of roadside backfill materials.

## 1. Introduction

Coal gangue and fly ash are the primary solid wastes generated by coal mining and coal-fired power plants. Large-scale stockpiling of these wastes not only occupies substantial land resources but may also cause contamination of water bodies, soil, and the atmospheric environment [1,2]. To improve solid-waste utilization and mitigate environmental impacts in mining areas [3,4], coal gangue and fly ash have increasingly been used to produce cemented gangue backfill. This material has been widely applied in goaf filling and roadway support [5,6]. However, similar to conventional concrete, such cemented backfill materials generally exhibit high brittleness and low ductility [7], and are prone to sudden brittle failure, which severely restricts their engineering application under high-intensity mining disturbances [8,9]. Inspired by the design concept of fiber-reinforced concrete, the incorporation of steel fibers has become an effective technical approach for enhancing the toughness and crack resistance of backfill materials [10,11].

Numerical simulation techniques offer irreplaceable advantages in investigating material failure mechanisms, particularly in revealing the formation mechanisms of macroscopic mechanical behavior from a mesoscopic perspective. Particle Flow Code (PFC), based on the discrete element method, is capable of simulating the mechanical response and crack evolution of particle assemblies, and has been widely applied in fracture analyses of brittle materials such as rock and concrete. For example, Liu et al. [12] established a two-dimensional PFC model for cemented backfill to simulate the influence of curing time on mesoscopic parameters; their study systematically examined the evolution of bond strength and stiffness at different curing ages, providing mesoscopic insight into the long-term strength prediction of backfill materials. Li et al. [13] investigated the effects of gangue particle size on the compressive deformation and crushing behavior of goaf backfill, demonstrating that larger aggregate sizes tend to form more pronounced force chain skeletons but also experience earlier particle breakage, thereby intensifying strain-softening behavior. Zhou et al. [14] simulated the rupture mechanism of microcapsules in concrete, highlighting the potential of PFC in mesoscopic damage analysis; their parametric study revealed the effects of capsule wall thickness and matrix strength on rupture behavior, providing theoretical support for the design of self-healing concrete.

In the field of fiber-reinforced materials, Zheng et al. [15] analyzed the mechanical properties and crack propagation behavior of steel fiber-reinforced concrete. Their model accurately captured the fiber-bridging effect and multiscale crack evolution, validating the applicability of PFC in simulating fiber-reinforced composites. Luis Felipe dos Santos Ribeiro [16] conducted PFC modeling of the mechanical behavior and crack evolution of fiber-reinforced concrete, with particular emphasis on the effects of fiber orientation and random distribution on tensile performance, offering valuable references for the incorporation of steel fibers into CGB. Xiong et al. [17] investigated crack propagation and damage mechanisms in fiber-reinforced cemented backfill, systematically comparing crack growth rates and damage accumulation processes under different fiber contents, and clarifying the critical role of fibers in the brittle-to-ductile transition. In addition, Xu et al. [18] explored the strengthening effects of molybdenum tailings and basalt fiber-modified composites on reinforced concrete, demonstrating that fibers effectively mitigate the brittle behavior of tailings sand and providing guidance for fiber modification in civil engineering applications. Jiao et al. [19] studied the synergistic effects of basalt fibers and polymers through mechanical testing and discrete element simulations, revealing that fibers primarily enhance compressive and flexural strength, whereas polymers significantly influence fiber–matrix interfacial properties and splitting tensile strength. Gu et al. [20] systematically calibrated and validated discrete element models of fiber-reinforced cement-treated aggregates, proposing an inversion approach for mesoscopic parameters based on macroscopic experimental data, which substantially improved model prediction accuracy. Su et al. [21] investigated the mechanical and shrinkage properties of polymer-modified cement mortar reinforced with recycled scrap tire steel fibers, and established a two-dimensional DEM model to elucidate the micromechanical mechanisms by which fibers suppress macroscopic cracking and enhance material toughness.

**Figure 1:**
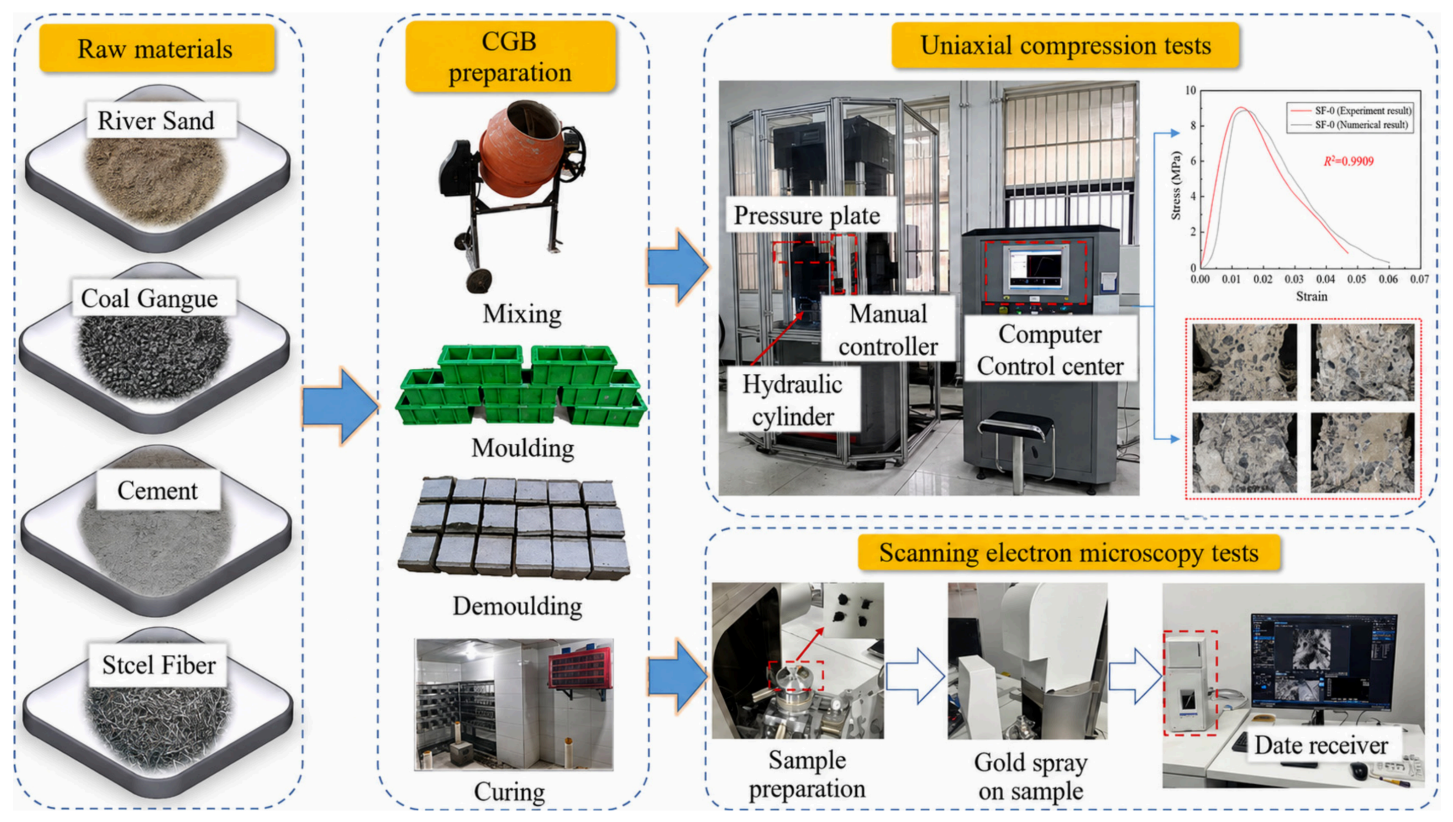
Experimental procedure and workflow of the study.

Although PFC has been extensively applied to simulations of conventional cemented backfill, systematic investigations on fiber-reinforced cemented backfill remain relatively limited. By constructing a fiber–matrix mesoscopic model using PFC, key information such as fiber bridging behavior during loading, crack propagation paths, force chain evolution, and pore structure development can be explicitly captured, thereby providing a theoretical basis for predicting damage evolution and optimizing material performance. Accordingly, this study establishes a mesoscopic numerical model of CGB that integrates realistic aggregate gradation and random distribution of steel fibers. Mesoscopic parameters are determined through inverse analysis, and uniaxial compression processes are simulated for different steel fiber volume fractions (0%, 0.4%, 0.8%, and 1.2%) (the detailed research framework is shown in Figure 1). The effects of steel fiber content on the macroscopic mechanical properties and mesoscopic damage mechanisms of CGB are systematically analyzed, with the aim of elucidating the reinforcement mechanism and identifying the optimal fiber content range.

## 2. Materials and Methods

### 2.1. Experimental Materials

The raw materials used for preparing the CGB specimens in this study included P.O. 42.5 ordinary Portland cement (OPC; Xuzhou Zhonglian Cement Co., Ltd., Xuzhou, China), coal gangue (CG; Xuzhou Mining Group Co., Ltd., Xuzhou, China), river sand (RS; Xuzhou Haoye Building Materials Co., Ltd., Xuzhou, China), laboratory tap water (WA), and steel fibers (SF; Lianyungang Weicheng New Materials Co., Ltd., Lianyungang, China) [22]. OPC was employed as the cementitious binder [23], with major mineral constituents including plagioclase, gypsum, calcite, potassium feldspar, and kaolinite, and chemical compositions dominated by CaO, SiO_2_, and Al_2_O_3_. The coarse aggregate (CG) consisted of coal gangue with a continuous particle size gradation of 5–10 mm, containing quartz, illite, kaolinite, and pyrite, and was mainly composed of SiO_2_, Al_2_O_3_, and CaO. The aggregate gradation used in this study is presented in Table 1. The fine aggregate (RS) was river sand with particle sizes smaller than 4.75 mm, primarily composed of quartz, feldspar, calcite, and illite, with SiO_2_ and Al_2_O_3_ as the dominant chemical components. In addition, steel fibers with an aspect ratio of 38 (25 mm × 0.65 mm) were incorporated to enhance the crack resistance and toughness of the CGB. The steel fibers used in this study were manufactured from cold-drawn low-carbon steel wire. They had a length of 25 mm, a diameter of 0.65 mm, an aspect ratio of 38, and a density of 7850 kg/m^3^. The nominal tensile strength, elastic modulus, and elongation at break were 1100 MPa, 200 GPa, and 3.5%, respectively. The proportions of each aggregate fraction and the detailed chemical compositions are presented in Figure 1 and Table 2.

### 2.2. Specimen Preparation

CGB specimens were prepared using a mass ratio of coal gangue, river sand, cement, and water of 2:4:1:1.235, as listed in Table 3. The solid constituents, including coal gangue, river sand, and cement, accounted for approximately 85% of the total mass. The specimens were cast as standard cubes with dimensions of 100 mm × 100 mm × 100 mm. Four groups were designed according to steel fiber volume fractions of 0%, 0.4%, 0.8%, and 1.2%, respectively, with the detailed mix proportions listed in Table 3. The four steel fiber volume fractions were selected to establish a controlled and sufficiently broad dosage gradient for evaluating the fiber-content-dependent mechanical response of CGB. The SF-0 group was used as the reference group to characterize the behavior of the unreinforced material. The volume fractions of 0.4% and 0.8% represented low and moderate fiber dosages, respectively, and were used to investigate the progressive development of the fiber-bridging and crack-arresting effects. A relatively high volume fraction of 1.2% was included to determine whether excessive fiber addition would adversely affect fiber dispersion and matrix compactness. Moreover, the uniform interval of 0.4% facilitated a systematic comparison of the mechanical and mesoscopic evolution characteristics among the different groups. Specimen codes were denoted by a combination of letters and numbers; for example, “SF-0.4” indicates a steel fiber volume fraction of 0.4%.

The weighed raw materials were placed in a mixing container and mixed while water was gradually added. The freshly mixed material was then cast into standard cubic molds. Three specimens were prepared for each group. After being stored indoors for 24 h, the specimens were demolded and cured for 28 days under standard conditions at a temperature of 20 ± 2 °C and a relative humidity of 95% ± 1%.

### 2.3. Uniaxial Compression Test

Uniaxial compression tests were conducted on cubic CGB specimens measuring 100 mm × 100 mm × 100 mm using a WDW-100 high-precision electronic universal testing machine (Jinan Chenda Testing Machine Manufacturing Co., Ltd., Jinan, Shandong, China) with a maximum load capacity of 100 kN. The specimens were loaded at a constant displacement rate of 0.4 mm/min until failure. The uniaxial compression test was terminated when the stress of the CGB specimen decreased to 70% of its peak strength. Three replicate specimens were tested for each mix proportion, resulting in a total of 12 uniaxial compression tests.

### 2.4. SEM Analysis

The microstructural characteristics of the CGB specimens were examined using a Zeiss EVO 18 scanning electron microscope (Carl Zeiss AG, Oberkochen, Germany). Prior to testing, the specimens were dried in an oven at 40 °C, and intact subspecimens were cut from the interior of the specimens. The subspecimens were then mounted onto specimen holders using conductive adhesive, followed by gold sputtering twice on the specimen surfaces using a vacuum coating apparatus to enhance electrical conductivity. Finally, the mounted specimens were placed in the SEM chamber for observation. The main operating parameters of the SEM included an accelerating voltage of up to 20 kV, a maximum magnification of 10,000×, and a resolution of 3 nm. The detailed experimental procedure is illustrated in Figure 1.

### 2.5. PFC2D Numerical Simulation

Numerical simulations were conducted using Particle Flow Code in Two Dimensions (PFC2D), version 6.0 (Itasca Consulting Group, Inc., Minneapolis, MN, USA). The numerical procedure included model construction, steel fiber generation, mesoscopic parameter calibration, uniaxial compression loading, and extraction of force chain, particle displacement, velocity, and crack evolution data.

#### 2.5.1. Selection of the Particle Bonding Model

When using PFC2D to simulate cemented backfill materials, it is necessary to account for the influence of cementitious binders, such as cement mortar, on the interactions between particles [24]. The parallel bond model is particularly well suited for this purpose and exhibits distinct advantages over other bonding models [25]. In this model, a virtual disk with radius r is established at the contact point, within which a set of parallel springs with constant stiffness is uniformly distributed, where the mean value of r represents the radius of the bonded interface. These parallel springs are capable of transmitting not only normal and shear forces between particles but also bending moments, which is highly consistent with the mechanical behavior of the cementitious matrix within cemented backfill materials.

#### 2.5.2. Establishment of the CGB Model and Calibration of Mesoscopic Parameters

**Figure 2:**
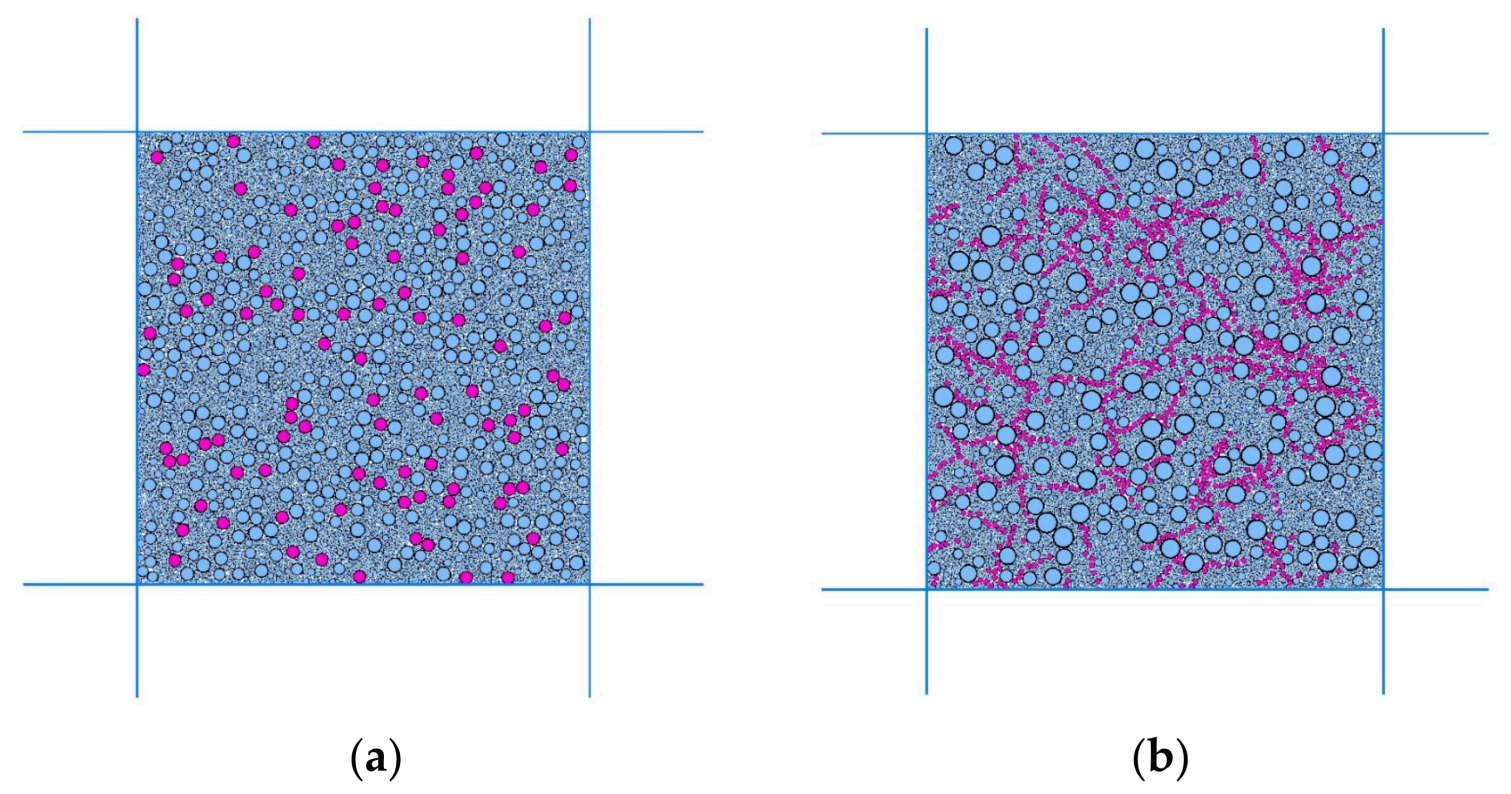
Construction of the CGB particle flow simulation model: (**a**) Particle distribution of the cemented gangue backfill. (**b**) Steel fiber distribution.

A two-dimensional numerical model with dimensions of 100 mm × 100 mm was established using rigid walls to reproduce the geometry of the laboratory specimens. Coal gangue and river sand particles were generated within the computational domain using the radius-expansion method according to the actual aggregate gradation. Because particles with insufficient contacts may remain suspended in the cementitious matrix and introduce unrealistic porosity and unstable force transmission, particles having fewer than three contacts with neighboring particles were defined as floating particles and removed from the model. After the floating particles were eliminated, the model contained a total of 10,980 particles, as shown in Figure 2.

The cemented matrix was represented using the parallel bond model, which allows the transmission of normal force, shear force, and bending moment between contacting particles. The initial mesoscopic parameters were assigned according to the values listed in Table 4. Steel fibers with volume fractions of 0%, 0.4%, 0.8%, and 1.2% were randomly distributed within the model using FISH programming. To represent the geometry and bending behavior of the steel fibers, fiber templates were initially generated as clumps composed of multiple pebbles. The positions of the individual pebbles were recorded, after which balls were generated at the same locations to form discrete fiber elements, and the original clumps were deleted [26,27]. During the subsequent equilibration process, interactions between the fiber elements and surrounding particles allowed the fibers to develop bent configurations.

The model was cycled until mechanical equilibrium was achieved. Mesoscopic parameters were then calibrated through inverse analysis by iteratively comparing the simulated and experimental stress–strain curves, peak strengths, post-peak responses, and failure modes [28,29,30]. The parameters were adjusted until satisfactory agreement between the numerical and experimental results was obtained.

After calibration, uniaxial compression was applied through the upper and lower rigid walls under displacement-controlled loading, while the lateral boundaries remained unconfined. Numerical simulations were conducted for each steel fiber volume fraction to evaluate the influence of fiber content on the mechanical and mesoscopic behavior of CGB. During loading, the stress–strain response, force chain distribution, particle displacement and velocity fields, and tensile and shear bond-break events were continuously recorded. These outputs were subsequently used to analyze the force transmission characteristics, deformation behavior, and crack initiation, propagation, and coalescence processes of the specimens.

## 3. Results and Discussion

### 3.1. Validation of the PFC2D Model Based on Stress–Strain Responses and Failure Modes

**Figure 3:**
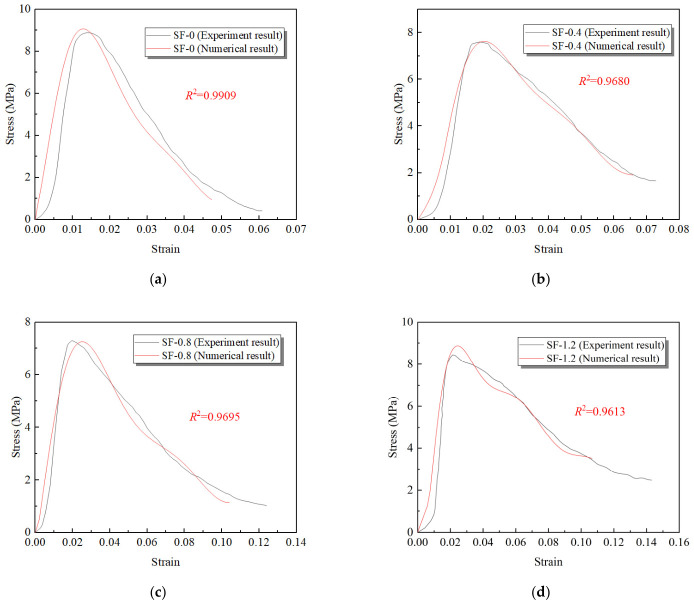
Comparison of stress–strain curves between laboratory tests and numerical simulations: (**a**) SF-0. (**b**) SF-0.4. (**c**) SF-0.8. (**d**) SF-1.2.

Figure 3 presents a comparison between the stress–strain curves obtained from laboratory tests and those predicted by the numerical models. In PFC2D, the macroscopic mechanical behavior of a model is governed by microscopic processes such as particle contact, bonding, and sliding [31,32]. As CGB is a particulate medium, its mesoscopic parameters cannot be directly measured, making inverse modeling essential for parameter determination. Through repeated calibration of the mesoscopic parameters, close agreement was achieved between the PFC-simulated and experimentally measured stress–strain curves, with correlation coefficients (R^2^) exceeding 0.96 in all cases.

The simulated and experimental peak strengths agreed closely, although minor differences remained between the corresponding stress–strain curves. These differences can be attributed to the following factors: during the initial loading stage, numerous pre-existing microcracks and pores within the CGB specimens are gradually compacted, resulting in a concave-downward segment in the experimental stress–strain curve. In contrast, the PFC2D model is generated by particles connected through an initially undamaged parallel bond model, in which the compaction stage is not pronounced. Nevertheless, the numerical model is capable of reproducing the elastic stage, plastic deformation stage, and failure stage of the specimens.

Under uniaxial compression, the failure modes observed in laboratory tests and numerical simulations are in good agreement. With increasing load, tensile and shear cracks progressively develop and coalesce, ultimately leading to an oblique shear failure plane inclined at a certain angle to the loading direction. The calibrated key mesoscopic parameters of the particle model corresponding to the final fitting results are summarized in Table 4.

### 3.2. Microstructural Analysis of CGB

**Figure 4:**
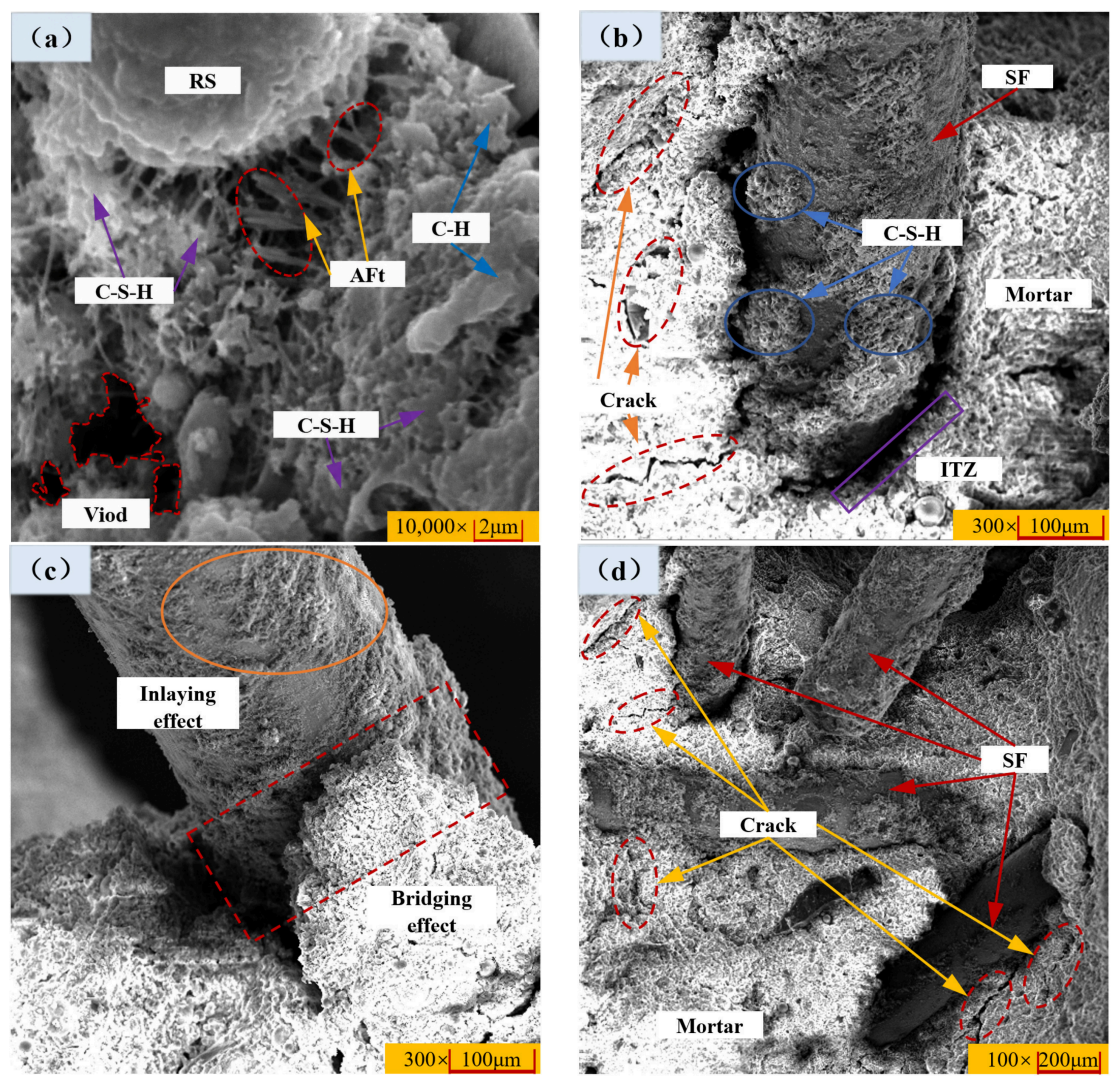
SEM micrographs of CGB with different steel fiber volume fractions. (**a**) 0%, showing hydration products, river sand particles, and internal voids; (**b**) 0.5%, showing the steel fiber–matrix interfacial transition zone and local cracks; (**c**) 1.0%, showing the fiber inlaying and crack-bridging effects; and (**d**) 1.5%, showing the distribution of steel fibers and their bridging effect across cracks.

To further elucidate the mechanisms by which steel fibers influence the microstructure of CGB, scanning electron microscopy (SEM) was employed to compare the micro-morphological characteristics of specimens with and without steel fiber incorporation. As shown in Figure 4a, the CGB without steel fibers is mainly composed of typical cement hydration products, including reticular or flocculent calcium silicate hydrate (C–S–H) gel, needle-like ettringite (AFt), and plate-like calcium hydroxide (C–H). These hydration products interweave to form a skeletal structure, among which the C–S–H gel [33] serves as the primary contributor to the strength of the backfill by bonding aggregate particles and filling internal pores, thereby enhancing overall compactness. However, due to moisture evaporation and shrinkage during the hardening process of cement-based materials, numerous initial pores and microcracks are generated, which become critical factors triggering instability and failure under loading.

In the CGB specimens incorporating steel fibers (Figure 4b–d), the microstructure exhibits significant improvement. After UCS testing, the steel fibers (SF) remain intact, with a small amount of matrix debris adhering to their surfaces (Figure 4b), indicating the formation of a well-developed interfacial transition zone (ITZ) between the fibers and the cementitious matrix. The presence of this ITZ enables steel fibers to dissipate energy through pull-out and sliding during loading, thereby effectively suppressing the propagation of macroscopic cracks. Moreover, the irregular geometry of steel fibers allows them to be tightly wrapped by hydration products, generating a pronounced interlocking effect [34] that enhances the fiber–matrix bonding strength (Figure 4c). This interlocking effect not only increases the interfacial bonding force but also further improves the compactness of the matrix. During the microcrack initiation stage, steel fibers act as crack bridges, delaying crack propagation and transforming the originally brittle failure mode into a more ductile failure behavior.

When the steel fiber content is excessively high (Figure 4d), fibers tend to agglomerate and create inter-fiber voids, which hinder effective bonding with the matrix and ultimately reduce the toughening efficiency [35]. Although some fibers remain embedded in the cement mortar, their non-uniform distribution weakens the overall synergistic effect, leading to a deterioration in mechanical performance. These observations demonstrate that an appropriate steel fiber content can significantly enhance the compressive strength, ductility, and crack resistance of CGB by optimizing its microstructure through the aforementioned mechanisms, whereas excessive fiber incorporation may produce adverse effects.

### 3.3. Numerically Simulated Mesoscopic Evolution of CGB

Based on the experimentally observed mechanical behavior, failure modes, and microstructural characteristics, the calibrated PFC2D models were further used to interpret the mesoscopic damage evolution of CGB with different steel fiber contents. The results presented in this section were obtained from the calibrated PFC2D numerical models. The force chain distributions, particle displacement and velocity fields, and crack evolution processes were extracted at selected loading stages to investigate the mesoscopic failure behavior of CGB with different steel fiber contents.

#### 3.3.1. Force Chain Distribution

**Figure 5:**
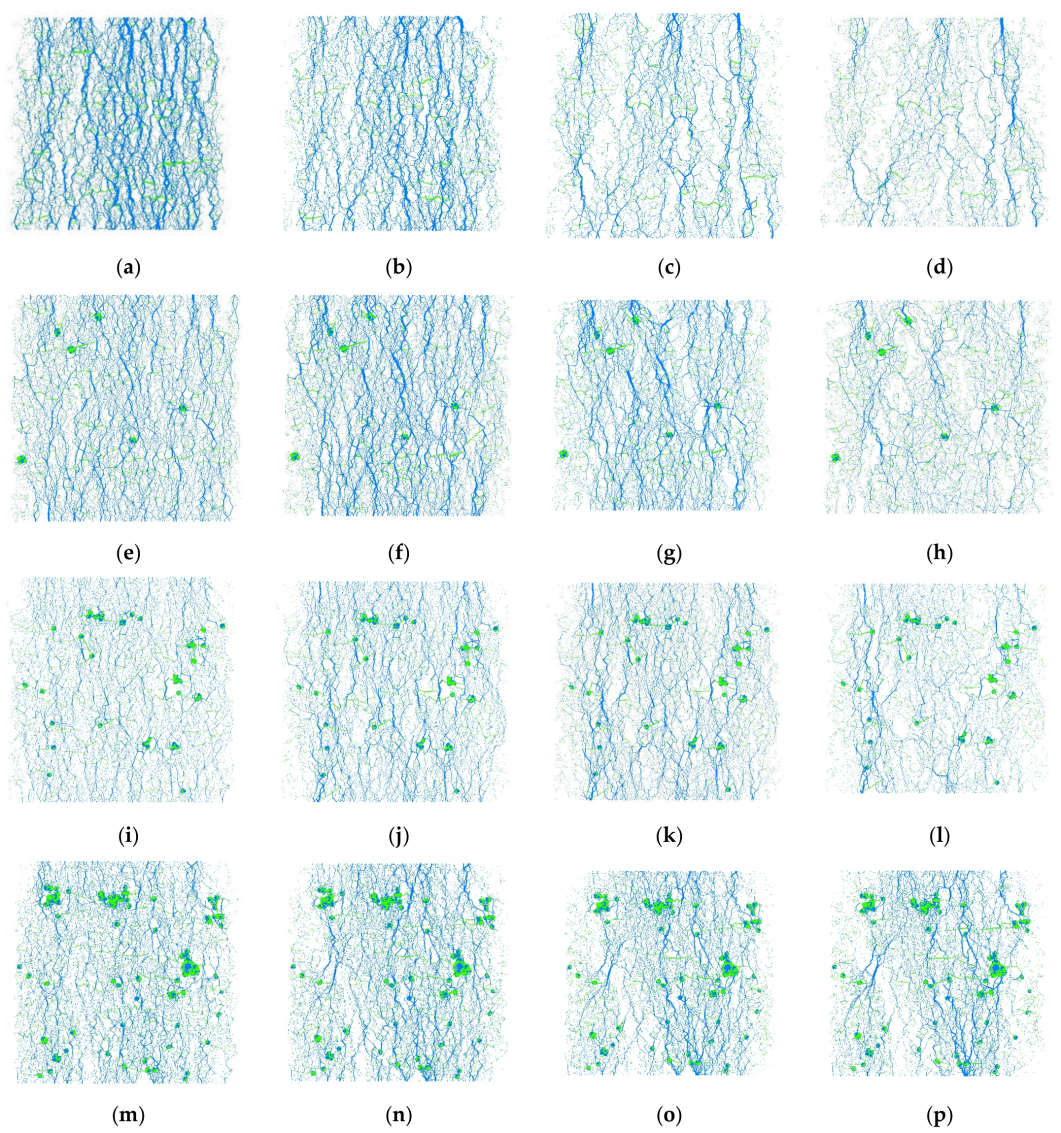
Force chain distributions obtained using PFC2D for CGB specimens with different steel fiber contents: (**a**) SF-0: 80% pre-peak stress. (**b**) SF-0: peak stress. (**c**) SF-0: 80% post-peak stress. (**d**) SF-0: complete failure. (**e**) SF-0.4: 80% pre-peak stress. (**f**) SF-0.4: peak stress. (**g**) SF-0.4: 80% post-peak stress. (**h**) SF-0.4: complete failure. (**i**) SF-0.8: 80% pre-peak stress. (**j**) SF-0.8: peak stress. (**k**) SF-0.8: 80% post-peak stress. (**l**) SF-0.8: complete failure. (**m**) SF-1.2: 80% pre-peak stress. (**n**) SF-1.2: peak stress. (**o**) SF-1.2: 80% post-peak stress. (**p**) SF-1.2: complete failure.

A boundary servo-control mechanism was employed to model the particle flow, and four representative points on the stress–strain curve—80% of the pre-peak stress, peak stress, 80% of the post-peak stress, and complete failure—were selected as reference states to analyze the evolution of force chains. Figure 5 shows the evolution of force chains in the CGB model during uniaxial compression. Blue and green chains represent compressive and tensile forces, respectively, and chain thickness indicates force magnitude. Owing to the non-uniform particle size distribution, the applied axial load is not evenly transmitted to all particle elements throughout the loading process. Instead, pronounced “skeleton force chain” structures develop around larger particles.

For the fiber-free specimen (SF-0 group), the contact forces are relatively uniformly distributed during the initial loading stage (80% pre-peak stress). As the load increases, the force chain intensity near the loading platens increases, causing high contact forces at the specimen boundaries to exceed the strength threshold of the model and generate discrete microcracks. Stress subsequently concentrates at the tips of these microcracks, leading to premature failure. At the peak loading stage, in the three models incorporating steel fibers, the “skeleton force chains” are predominantly aligned along the axial stress direction and initially play a load-bearing role. The introduction of steel fibers causes the “skeleton force chains” around particles to be preferentially disrupted, resulting in particle crushing, collision, and infilling. When steel fibers are distributed between two cracks, their bonding and bridging effects become evident, effectively suppressing the development of tensile cracks within the specimen.

During the post-peak stage, the force chain network evolved continuously, and the load-bearing skeleton force chains underwent substantial redistribution. Similar force chain redistribution has also been reported in previous PFC studies of cemented backfill under axial compression [36]. In the initial state, force chains are mainly concentrated around coarse gangue aggregate particles, exhibiting sparse distribution and relatively low intensity. With increasing axial pressure, pores are gradually compacted, inter-particle bonds are progressively broken, and particle positions are rearranged. Consequently, the intensity of strong force chains decreases, while that of weaker force chains increases, leading to a more stable overall force chain network.

A comparison of force chain distributions under different steel fiber contents reveals that, with increasing steel fiber volume fraction, the force chain connections in the pre-peak stage become more stable, while the skeleton force chains gradually become thinner, indicating a progressive reduction in local force intensity and compressive strength. Under continued axial loading, force chain networks eventually rupture, corresponding to the macroscopic failure of the specimen. Overall, the incorporation of steel fibers markedly alters the distribution and evolution of force chains, enhances the tensile resistance and toughness of the material, retards crack propagation, and thereby improves the overall mechanical performance of the CGB.

#### 3.3.2. Displacement Field Distribution

**Figure 6:**
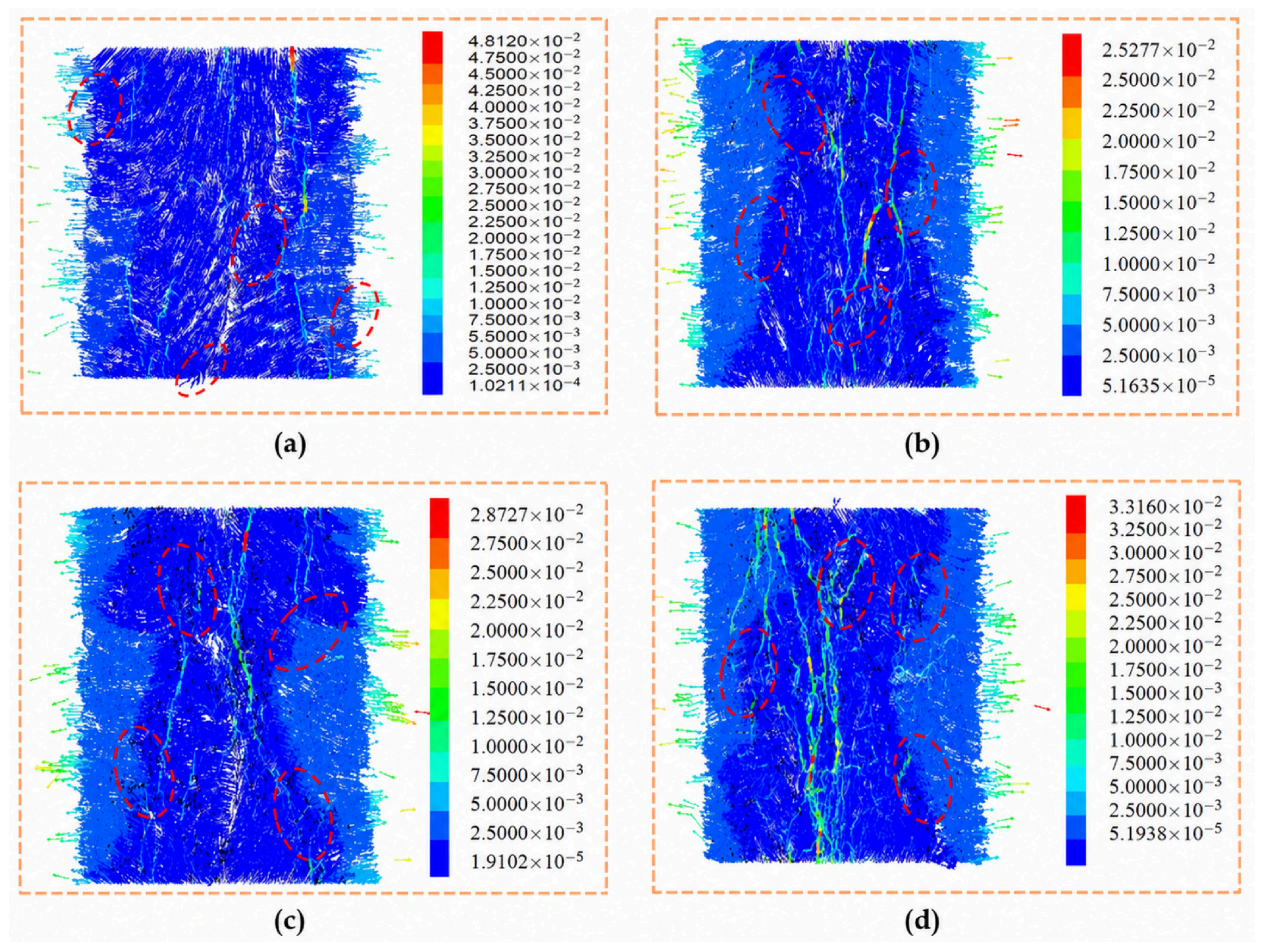
Displacement field distributions of CGB specimens after complete failure: (**a**) SF-0. (**b**) SF-0.4. (**c**) SF-0.8. (**d**) SF-1.2.

Figure 6 illustrates the displacement field distributions of CGB specimens with different steel fiber contents after complete failure. Particle velocity and displacement fields have been widely used to identify localized deformation and crack development in particle-flow simulations [37]. In the present study, the displacement fields were analyzed to clarify the failure characteristics of CGB specimens with different steel fiber contents. For the fiber-free specimen (SF-0 group), the displacement fields of particles in the upper and lower regions are highly disordered, with particles moving uncontrollably toward both lateral sides. This unrestricted motion promotes crack propagation and coalescence into a dominant macrocrack, while stress concentration zones generate localized microcracks (indicated by the black regions in the figure).

In contrast, for specimens incorporating steel fibers (SF-0.4, SF-0.8, and SF-1.2 groups), although the particle displacement fields also exhibit a certain degree of disorder, the majority of particles migrate toward the unconfined lateral surfaces and form relatively stable displacement planes (highlighted by the red circles in the figure), indicating the presence of through-going cracks. The incorporation of steel fibers enhances inter-particle interactions, bridges developing cracks, and retards their propagation, thereby significantly improving the overall crack resistance of the material.

By examining the evolution of microcracks, the macroscopic failure patterns of the specimens can be inferred. The presence of steel fibers alters particle motion trajectories, promotes the formation of organized displacement planes, and enhances the overall structural stability and tensile resistance of the material. These findings offer practical implications for the design of high-performance roadside backfill materials.

#### 3.3.3. Velocity Field Distribution

**Figure 7:**
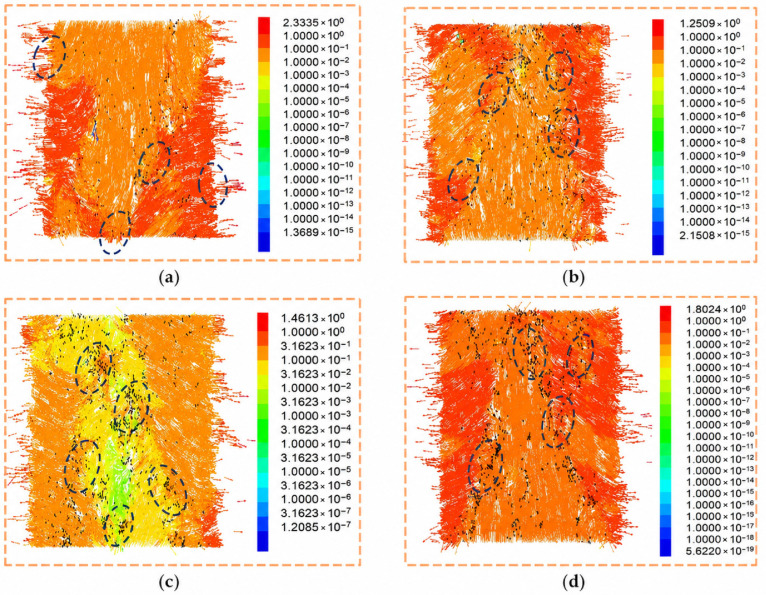
Velocity field distributions of CGB specimens after complete failure: (**a**) SF-0. (**b**) SF-0.4. (**c**) SF-0.8. (**d**) SF-1.2.

Figure 7 shows the velocity field distributions of CGB specimens with different steel fiber contents after complete failure. At the mesoscopic scale, the overall velocity directions of particles in the model are generally consistent with the displacement field patterns, while localized disordered regions are mainly concentrated in areas where microcracks develop. Comparison with experimental observations indicates that the abnormal regions of particle velocity and displacement directions correspond closely to the locations of macroscopic failure cracks, demonstrating that the numerical simulations effectively reproduce the crack evolution and failure modes observed in laboratory tests.

In Figure 7, the arrows denote the movement directions of spherical particles, and it can be observed that particle displacement directions are generally perpendicular to the crack propagation paths. With increasing steel fiber volume fraction, the inter-particle bonding effect is strengthened, resulting in more stable force chain structures under compression and a reduced tendency for force chain breakage. Consequently, both the particle displacement and velocity fields become more orderly, and the overall resistance to deformation is enhanced. From a mesoscopic perspective, these results further confirm the reinforcing effect of steel fibers on the crack resistance of CGB.

#### 3.3.4. Crack Initiation and Fracture Evolution

**Figure 8:**
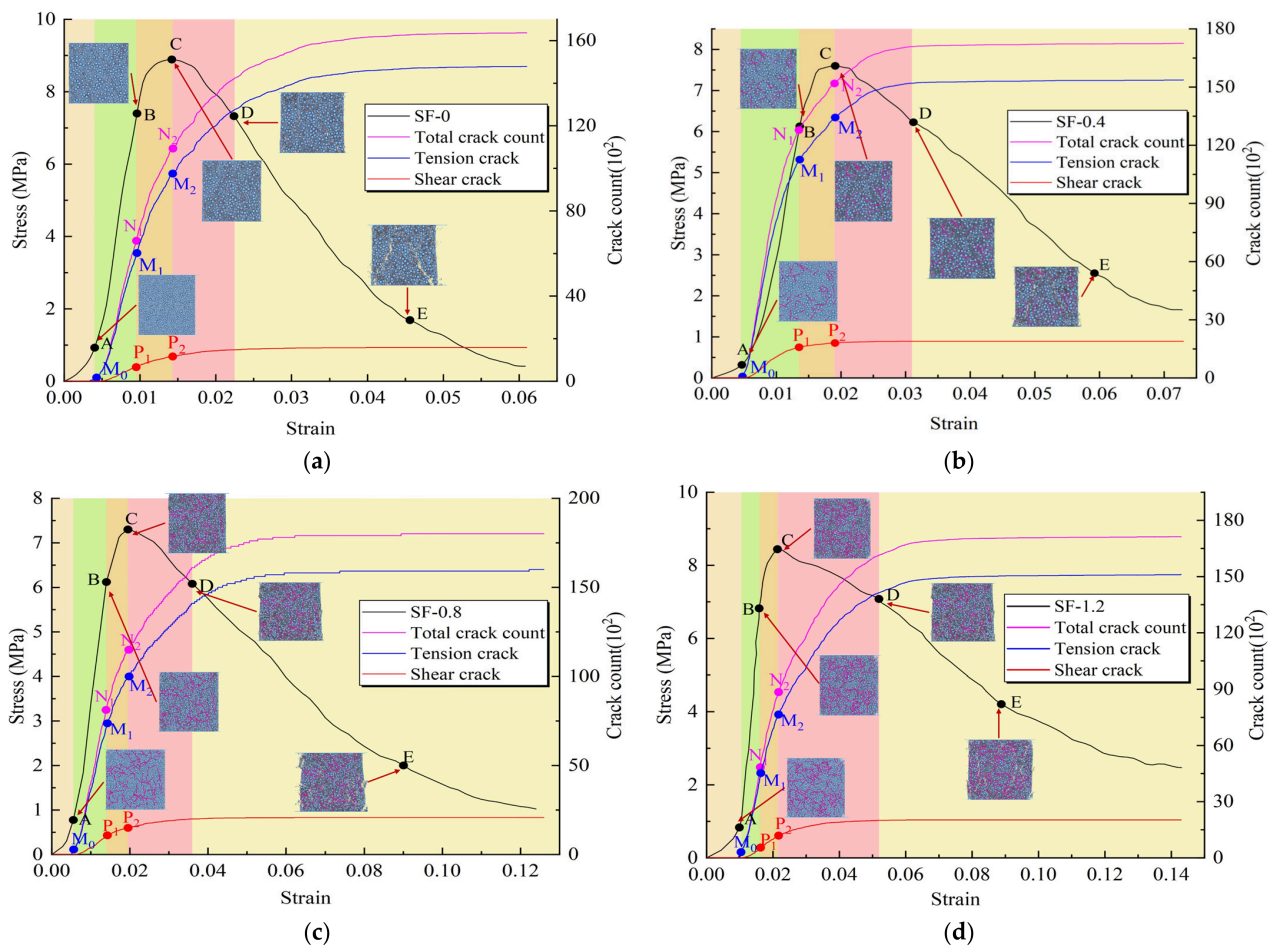
Crack evolution obtained from PFC2D numerical simulations for CGB specimens with different steel fiber contents: (**a**) SF-0. (**b**) SF-0.4. (**c**) SF-0.8. (**d**) SF-1.2.

Following the bond-break-based crack identification approach commonly used in previous PFC studies [38], the built-in FISH functions were used to record crack initiation, propagation, and coalescence during uniaxial compression. Particular attention was given to the evolution of the stress–strain relationship and the numbers of total cracks, tensile cracks, and shear cracks under different steel fiber contents (Figure 8). The crack evolution results presented in Figure 8 were obtained from the calibrated PFC2D numerical simulations. Crack initiation, propagation, and coalescence were tracked using the built-in FISH functions. Based on the characteristics of crack number evolution, the progressive failure of CGB was divided into five distinct stages, and the characteristic stress levels and crack evolution mechanisms at each stage were clarified as follows.

(1) Pore compaction stage.

The stress–strain curve exhibits a concave-upward shape, as initial micro-pores and microcracks within the material close under external loading, accompanied by slight volumetric contraction. At this stage, the total number of cracks is extremely small and consists exclusively of shear cracks. The crack evolution curve is relatively smooth with a small slope. The influence of steel fiber content at this stage is limited: low fiber content (SF-0.4) can slightly reduce porosity, whereas high fiber content (SF-1.2) may introduce local heterogeneity in pore distribution due to non-uniform fiber dispersion. Overall, the effect remains insignificant.

(2) Linear elastic deformation stage.

The stress–strain relationship follows Hooke’s law and exhibits a linear trend, with characteristic point A corresponding to the elastic limit. The total crack evolution curve becomes concave upward, and after the crack initiation stress point (M0), the curve slope increases, indicating an elevated crack growth rate. Tensile cracks dominate this stage, accounting for more than 97% of the total cracks. After point P1, the growth rate of shear cracks slows down, while beyond point M1, the slope of tensile crack evolution further increases. Low to moderate steel fiber contents (SF-0.4–0.8) enhance the elastic modulus of the material, increase the slope of the stress–strain curve, and delay the onset of yielding (point B). In contrast, a high fiber content (SF-1.2), although increasing stiffness, may induce early plastic deformation due to fiber–fiber interactions or reduced matrix continuity, thereby shortening the plastic deformation interval.

(3) Plastic yielding stage.

The total crack evolution curve exhibits a sharp increase, and beyond point B, the number of tensile cracks rises abruptly, while shear cracks begin to appear. With continued crack accumulation, macroscopic damage develops until the peak stress σc is reached (corresponding to points N2, M2, and P2). After point P2, the growth of shear cracks approaches its maximum, whereas beyond point M2, the slope of tensile crack evolution decreases but crack initiation continues. An appropriate steel fiber content (SF-0.8) effectively disperses stress through the fiber-bridging effect, thereby reducing crack propagation rates. Conversely, excessive fiber content (SF-1.2) may intensify local stress concentration due to fiber agglomeration or interfacial weakening, accelerating crack propagation and reducing fracture resistance.

(4) Post-peak failure stage.

Point C corresponds to the peak stress, at which macroscopic failure occurs. Through-going cracks penetrate deeply into the cemented matrix and become fully connected, consistent with the numerical simulation results. The stress–strain curve exhibits strain-softening behavior, with stress decreasing as strain increases. Point D corresponds to 80% of the post-peak residual stress. At this stage, the “skeleton effect” of steel fibers becomes prominent, particularly in the SF-0.4–0.8 groups, where the fiber network helps maintain residual strength and ensures a certain load-bearing capacity after failure.

(5) Post-peak residual stage.

Although the internal structure of the material has been severely damaged, its overall integrity remains relatively intact. Beyond point D, cracks continue to develop and intersect, coalesce, and penetrate, eventually forming macroscopic crack or fracture planes. Crack evolution ceases at point E (complete failure), and the corresponding stress is defined as the residual strength. Compared with the fiber-free specimen (SF-0), fiber-reinforced specimens exhibit no obvious through-going cracks, reduced matrix spalling, and larger deformation at failure under axial loading. These specimens display typical plastic deformation, flow-like behavior, and axial compression–lateral dilation characteristics, indicating a significant improvement in ductility.

#### 3.3.5. Crack Rose Diagrams

**Figure 9:**
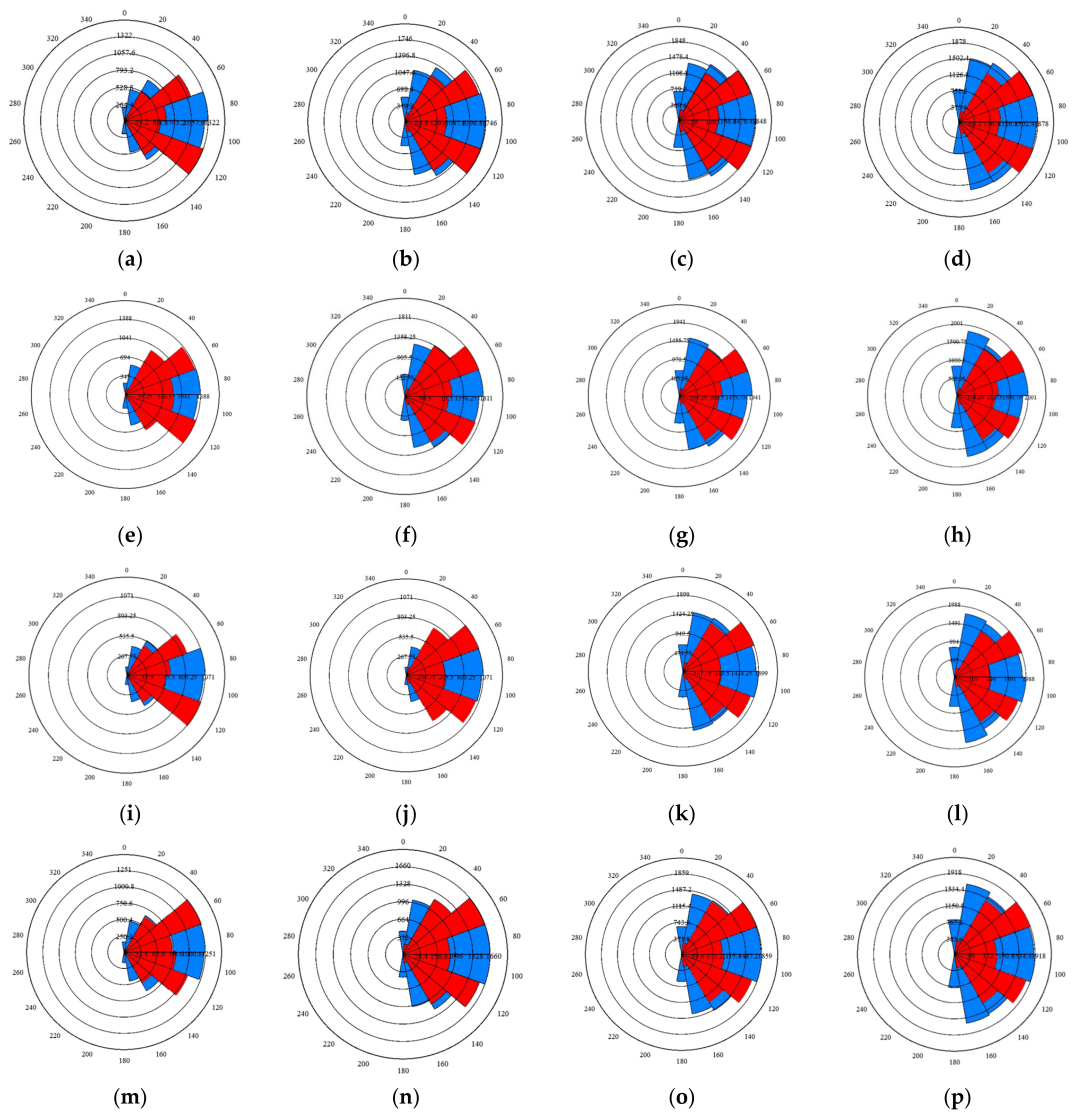
Rose diagrams of crack propagation in CGB with different steel fiber volume fractions: (**a**) SF-0: 80% pre-peak stress. (**b**) SF-0: peak stress. (**c**) SF-0: 80% post-peak stress. (**d**) SF-0: complete failure. (**e**) SF-0.4: 80% pre-peak stress. (**f**) SF-0.4: peak stress. (**g**) SF-0.4: 80% post-peak stress. (**h**) SF-0.4: complete failure. (**i**) SF-0.8: 80% pre-peak stress. (**j**) SF-0.8: peak stress. (**k**) SF-0.8: 80% post-peak stress. (**l**) SF-0.8: complete failure. (**m**) SF-1.2: 80% pre-peak stress. (**n**) SF-1.2: peak stress. (**o**) SF-1.2: 80% post-peak stress. (**p**) SF-1.2: complete failure.

Figure 9 presents the crack propagation rose diagrams of CGB specimens with different steel fiber volume fractions during uniaxial compression, revealing the evolution of crack types and quantities at various loading stages. It should be noted that the partially obscured horizontal numerical annotations in the rose diagrams are automatically generated by the software and are identical to the corresponding vertically displayed numerical annotations. Therefore, the unobscured vertical annotations can be used as reference, and the partial overlap does not affect the interpretation of the crack orientation or quantity. At the 80% pre-peak stage, cracks in all specimens are predominantly shear cracks and remain relatively limited in number. At this stage, pore closure dominates the deformation process, leading to slight volumetric contraction. A low steel fiber content (SF-0.4) slightly reduces porosity and results in a denser matrix, whereas a high fiber content (SF-1.2) may cause heterogeneous pore distribution.

At the peak stress stage, the number of cracks increases markedly, with tensile cracks becoming dominant and a substantial number of shear cracks also emerging. Low to moderate steel fiber contents (SF-0.4–SF-0.8) enhance the elastic modulus and yield strength of the material. In contrast, excessive fiber content (SF-1.2) may narrow the plastic deformation region and adversely affect toughness due to fiber–fiber interactions or reduced matrix continuity.

During the 80% post-peak stage, the total number of cracks continues to increase, although the growth rate slows. An appropriate fiber content (SF-0.8) effectively disperses stress and retards crack propagation, whereas a high fiber content (SF-1.2) promotes local stress concentration due to fiber agglomeration or interfacial weakening, thereby reducing fracture resistance. At the final failure stage, cracks penetrate through the material and coalesce into macroscopic fracture planes, and the load-bearing capacity decreases to its minimum level. The “skeleton effect” of steel fibers is observed at all fiber contents; however, moderate fiber contents (SF-0.4–SF-0.8) are more conducive to forming effective crack-arresting mechanisms, enabling the material to retain a certain residual load-bearing capacity after failure.

In contrast, the fiber-free material (SF-0) exhibits pronounced through-going cracks and more severe matrix spalling. Combined with the results shown in Figure 8, it is evident that steel fibers exert a significant influence on crack propagation at different loading stages. An appropriate fiber content provides consistent reinforcement across all stages, whereas excessive fiber content may reduce the overall material performance due to fiber–fiber interactions and interfacial deficiencies.

#### 3.3.6. Internal Pore Expansion

**Figure 10:**
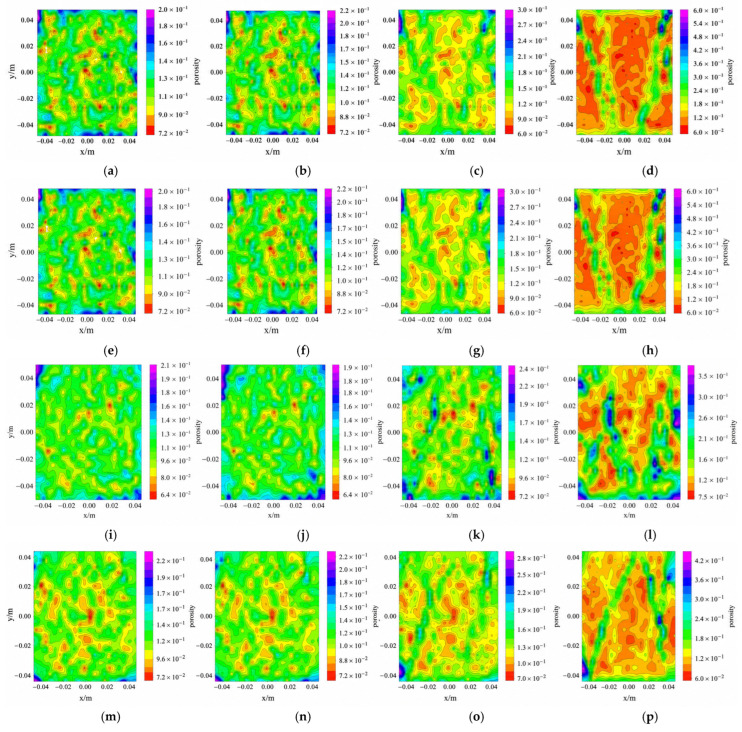
Porosity cloud maps of CGB with different steel fiber volume fractions: (**a**) SF-0: 80% pre-peak stress. (**b**) SF-0: peak stress. (**c**) SF-0: 80% post-peak stress. (**d**) SF-0: complete failure. (**e**) SF-0.4: 80% pre-peak stress. (**f**) SF-0.4: peak stress. (**g**) SF-0.4: 80% post-peak stress. (**h**) SF-0.4: complete failure. (**i**) SF-0.8: 80% pre-peak stress. (**j**) SF-0.8: peak stress. (**k**) SF-0.8: 80% post-peak stress. (**l**) SF-0.8: complete failure. (**m**) SF-1.2: 80% pre-peak stress. (**n**) SF-1.2: peak stress. (**o**) SF-1.2: 80% post-peak stress. (**p**) SF-1.2: complete failure.

As shown in the pore cloud maps in Figure 10, different steel fiber contents exert a pronounced influence on the pore structure and mechanical performance of CGB. In the absence of steel fibers (SF-0), the initial pore population is relatively large and unevenly distributed, resulting in a loose internal structure. With increasing stress, although some pores gradually close, poor pore connectivity promotes stress concentration, which facilitates the initiation of macroscopic cracks and ultimately leads to brittle failure [39]. Specifically, under the SF-0 condition, the number of pores decreases by approximately 25% in the pre-peak stage, but rapidly rebounds to about 10% higher than the initial level in the post-peak stage.

At a low fiber content (SF-0.4), an appropriate amount of steel fibers contributes to reducing porosity and making the matrix more compact. The interfacial transition zone (ITZ) between fibers and the matrix effectively disperses stress and suppresses the propagation of microcracks. Consequently, during the early loading stage, pores close more rapidly, and the pore number decreases by approximately 33.3%, resulting in a more uniform internal structure. Under this condition, the material exhibits improved compressive strength and toughness, while crack development is effectively restrained.

When a moderate fiber content is adopted (SF-0.8), steel fibers form a favorable interlocking effect within the matrix, further enhancing the fiber–matrix bonding performance. Particularly at peak stress, local stress concentration within pores induces plastic deformation of pore walls and the extension of microcracks, leading to an increase in pore size. Meanwhile, improved pore connectivity facilitates stress redistribution, thereby delaying the initiation of macroscopic cracks. At this stage, pore closure reaches 43.8%, and in the post-peak stage, the pore number increases by only 22.2%, demonstrating the superiority of this mix proportion in pore control.

However, at a high fiber content (SF-1.2), although the initial pore closure rate reaches the highest value of 46.7%, fiber–fiber interactions or reduced matrix continuity may result in heterogeneous pore distribution and intensified internal pore expansion. More microcracks develop along pore walls, accelerating localized damage and the failure process. In the post-peak stage, the pore number increases sharply by 62.5%, indicating that excessive fiber content may aggravate pore expansion due to fiber agglomeration or interfacial weakening, thereby degrading the overall material performance.

### 3.4. Multiscale Reinforcement Mechanism, Engineering Implications, and Limitations

The experimental, numerical, and microstructural results collectively reveal a multiscale reinforcement mechanism of steel fibers in CGB. At low to moderate volume fractions, particularly at 0.8%, the randomly distributed fibers bridge incipient cracks and transfer tensile stress across discontinuities. This bridging action reduces local stress concentration, promotes the redistribution of the force chain network, and delays the transition from isolated microcracks to a connected macroscopic fracture plane. Consequently, damage accumulation is retarded before the peak stress, while the fiber network continues to maintain structural integrity and load transfer after peak failure. This mechanism explains the simultaneous increase in peak strength, deformation capacity, and residual strength observed for the SF-0.8 specimens.

The PFC and SEM results provide complementary evidence for this interpretation. In the numerical model, the 0.8% fiber content produced a more homogeneous force chain network, reduced crack localization, and preserved a more stable load-bearing skeleton during the post-peak stage. At the microscale, the hydration products surrounding the fibers formed a relatively dense interfacial transition zone, which enhanced mechanical interlocking and enabled energy dissipation through fiber debonding, sliding, and pull-out. Therefore, the improved macroscopic behavior cannot be attributed solely to the intrinsic strength of the steel fibers; it results from the combined effects of crack bridging, stress redistribution, interfacial bonding, and post-peak load transfer.

In contrast, increasing the steel fiber volume fraction to 1.2% did not produce a further improvement. Excessive fibers tended to agglomerate and generate inter-fiber voids, thereby interrupting matrix continuity and weakening the effective fiber–matrix interface. These defects became preferential sites for stress concentration and crack initiation, which offset the beneficial bridging effect. This finding indicates that fiber reinforcement in CGB is governed by a balance between the number of effective crack-bridging fibers and the defects introduced by poor fiber dispersion. Accordingly, 0.8% should be regarded as the optimal steel fiber content within the investigated mixture system and dosage range, rather than as a universally applicable value.

From an engineering perspective, the post-peak behavior is particularly important for roadside backfill because the material must retain integrity and load-bearing capacity after local cracking under mining-induced disturbances. The increase in residual strength and the transition from brittle shear failure to a more ductile compressive–extrusion mode can reduce the likelihood of sudden loss of support capacity. The results also demonstrate that simply increasing the fiber dosage is not an effective design strategy. Controlling fiber dispersion and the fiber–matrix interfacial quality is equally important. The refined PFC2D approach developed in this study can be used as a numerical screening tool to compare fiber dosages and examine internal damage processes that are difficult to observe experimentally, thereby reducing the number of preliminary laboratory trials required for mixture optimization.

Nevertheless, the engineering interpretation of the results is subject to several limitations. The present conclusions are based on one aggregate gradation, one steel fiber geometry, a curing age of 28 days, and uniaxial compression conditions. In addition, the two-dimensional model simplifies the spatial orientation and interaction of fibers in the actual three-dimensional material. Future studies should examine different fiber geometries, aggregate gradations, curing ages, and loading paths, including cyclic and confined loading. Three-dimensional numerical modeling and field-scale validation are also required before the proposed dosage range is directly applied to specific engineering conditions.

## 4. Conclusions

In this study, numerical simulations of conventional uniaxial compression tests were conducted using the particle flow code PFC2D to investigate the mechanical behavior of CGB with different steel fiber volume fractions. By analyzing the evolution of force chains, displacement fields, velocity fields, and crack initiation and propagation characteristics, the macroscopic and mesoscopic failure behaviors and crack evolution laws of CGB during progressive damage were systematically examined. The main conclusions can be summarized as follows:

(1) The numerical simulation results show excellent agreement with laboratory test data, with an average error in peak strength of less than 5% and correlation coefficients (R^2^) exceeding 0.96 in all cases, confirming the reliability of the PFC2D model in simulating the mechanical behavior of CGB. The incorporation of steel fibers transforms the failure mode from typical shear-dominated failure to a ductile compressive–extrusion failure mode, significantly enhancing the deformation capacity of the material.

(2) Crack evolution analysis indicates that the failure process of CGB can be divided into five successive stages, and the steel fiber content exerts a pronounced influence on crack propagation in each stage. At a fiber volume fraction of 0.8%, the total number of cracks is reduced by approximately 40%, and the crack propagation rate decreases by 35%. In contrast, a higher fiber content of 1.2% produces an adverse effect, increasing the crack number by 15% and the propagation rate by 28%. These results demonstrate the existence of an optimal fiber content range (0.4–0.8%), beyond which the crack resistance of the material deteriorates.

(3) Microstructural analysis reveals that the steel fiber content directly affects the pore structure and interfacial characteristics of the material. At a fiber content of 0.8%, a dense ITZ is formed, with abundant C–S–H gel adhering to the fiber surfaces, and the pore closure rate reaches 43.8%. Conversely, at a fiber content of 1.2%, fiber agglomeration induces microcracking and interfacial debonding within the ITZ, resulting in a 62.5% increase in porosity. These microstructural changes exhibit a strong correlation with the degradation of macroscopic mechanical performance.

(4) Overall, the comprehensive performance of CGB is optimal at a steel fiber volume fraction of 0.8%, at which the peak strength and residual strength are increased by 23.7% and 40.2%, respectively, compared with the reference specimen. Excessive fiber incorporation (1.2%) leads to fiber agglomeration and weakened interfacial bonding, thereby reducing material performance. Therefore, precise control of steel fiber content is essential in enhancing the engineering performance of CGB.

## Figures and Tables

**Table 1:** Aggregate gradation of cemented gangue backfill.

Aggregate	CG	RS			
Particle size (mm)	5–10	2–4.75	1.5–2	1–1.5	<1
Percentage by mass (%)		3%	4%	9%	84%

**Table 2:** Chemical compositions of raw materials used in CGB.

Raw Materials	SiO_2_	Al_2_O_3_	K_2_O	Fe_2_O_3_	CaO	MgO	Na_2_O	Others
OPC	16.54	17.16	0.11	10.12	49.46	1.03	0.57	5.01
CG	40.36	24.3	0.94	4.97	22.7	-	-	6.73
RS	65.6	12.33	3.01	2.55	3.26	1.59	1.12	10.54

**Table 3:** Mix proportions of CGB specimens.

Group	OPC	CG	RS	SF	WA
SF-0	1	2	4	0	1.235
SF-0.4	1	2	4	0.2	1.283
SF-0.8	1	2	4	0.4	1.307
SF-1.2	1	2	4	0.6	1.345

**Table 4:** Mesoscopic mechanical parameters used in numerical simulations.

Detailed Examination of Mechanical Parameters		Value
Wall-Ball	Elastic modulus/Pa	10^9^
	Stiffness ratio	1.5
Ball-Ball	Density/(Kg/m^3^)	1880
	Clump friction	0.7
	Elastic modulus/Pa	10^8^
	Stiffness ratio	1.5
Parallel bonding model	Normal stiffness/(KPa/m)	5 × 10^7^
	Tangential stiffness/(KPa/m)	5 × 10^7^
	Parallel adhesion force/Pa	10^6^
	Parallel bonding tensile strength/Pa	10^6^
	Parallel bonding friction angle/°	30

## Data Availability

The original contributions presented in this study are included in the article. Further inquiries can be directed to the corresponding author.
